# Petal-Like Calcifications in Thyroid Nodules on Ultrasonography: A Rare Morphologic Characteristic of Calcification Associated With Aggressive Biological Behavior

**DOI:** 10.3389/fendo.2020.00271

**Published:** 2020-05-22

**Authors:** Qinghai Peng, Qi Zhang, Sijie Chen, Chengcheng Niu

**Affiliations:** Department of Ultrasound Diagnosis, Second Xiangya Hospital, Central South University, Changsha, China

**Keywords:** petal-like calcifications, conventional ultrasound, contrast-enhanced ultrasound, papillary thyroid carcinoma, lymph node metastasis

## Abstract

This study investigated a rare ultrasonographically detected thyroid petal-like calcification and its relationship with thyroid carcinoma and biological behavior. We described the clinical and ultrasonographical features of thyroid nodules with petal-like calcifications in 18 patients undergoing thyroid surgery and cervical lymph node dissection. All of the thyroid nodules with petal-like calcifications were papillary thyroid carcinomas (PTCs). Of the 18 patients, 13 (72.2%) had cervical central lymph node metastasis, and five (27.8%) had cervical lateral lymph node metastasis. Petal-like calcifications occurred in malignant thyroid nodules with a high incidence of lymph node metastasis, which may be a specific ultrasonographic feature associated with the aggressive biological behavior of PTC.

## Introduction

Calcifications are commonly detected by ultrasonographic images in thyroid nodules and could be classified into various patterns (1–6). Taki et al. classified calcifications into microcalcifications, intranodular coarse calcifications, peripheral calcifications, and calcified spots (7). Kim et al. further classified peripheral calcifications into annular-like peripheral calcifications and crescent-like peripheral calcifications (2, 3). Among these subtypes, microcalcifications are known to be highly associated with papillary thyroid carcinoma (PTC) (4, 5). Kobayashi et al. reported that, out of 941 PTC patients, 32.0% patients had microcalcifications, and, out of 407 thyroid nodules with microcalcification, 301 (74.0%) were PTC (4). Yin et al. reported that, among 339 thyroid nodules with microcalcification, 210 (61.9%) of them were PTC, and, of 312 PTC patients, 210 (63.1%) had microcalcifications (5). However, to our knowledge, there are no studies on the ultrasonographic features of petal-like calcifications in thyroid nodules. In this study, we investigated the detection of petal-like calcifications by ultrasound and their relationship with thyroid carcinoma and biological behavior.

## Methods

### Patients

A total of 18 patients with 18 nodules with petal-like calcifications that were detected with preoperative conventional ultrasound (US) and contrasted-enhanced ultrasound (CEUS) and underwent postoperative histopathologic analysis following resected hemithyroidectomy or total thyroidectomy from December 2016 to November 2019 were enrolled in this prospective study. Of the 18 patients, five underwent cervical central and lateral lymph node dissection, and 13 only underwent central lymph node dissection.

### Ultrasound Examination

**Figure 1:**
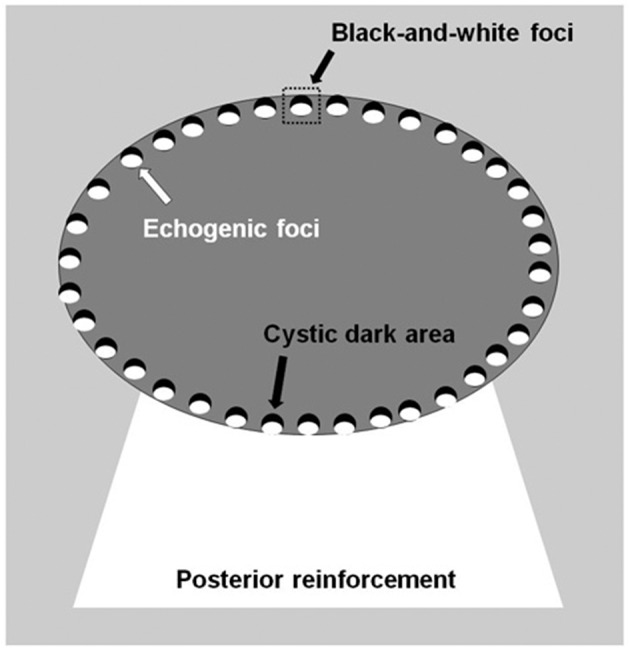
Diagram shows sonographic assessment of a petal-like calcification in thyroid nodules.

The thyroid nodules were imaged with a Siemens Acuson S3000 US scanner equipped with a 9L4 linear array transducer (Siemens Medical Solutions, Mountain View, CA, USA; transducer frequency: 4–9 MHz) and/or an 18L6 linear array transducer (6–18 MHz). Petal-like calcifications appeared as scattered hyperechogenic spots <2 mm in diameter around solid thyroid nodules with the appearance of flower petals and had a cystic-like dark area ahead of each hyperechogenic spot. The cystic dark area and hyperechogenic spot constituted black-and-white foci (Figure 1). The thyroid nodules with petal-like calcifications often showed acoustic posterior reinforcement. In addition to age, sex, and serum thyroid hormone, which included thyroid-stimulating hormone (TSH), free thyroxine, free triiodothyronine, thyroid peroxidases antibody (A-TPO), and thyroglobulin antibody (A-TG), the US features of the thyroid nodules were recorded, including tumor size, composition, shape, margin, echogenicity, posterior reinforcement, vascularity, and capsule contact with protrusion (8, 9). The US performance of the thyroid nodules was classified according to the Thyroid Imaging Reporting and Data System (TI-RADS) diagnostic classification by Kwak et al. (10).

CEUS was performed using contrast pulsed sequencing technology with a low mechanical index following intravenous injection of SonoVue microbubbles (Bracco, Italy). CEUS videos were recorded for at least 60 s with dedicated software (Contrast Dynamics, Mountain View, CA, USA). With respect to the surrounding thyroid parenchyma enhancement, the time-intensity curves (TICs) of the thyroid nodules with regions of interest (ROIs) were acquired, and CEUS features were classified (8, 9), including enhancement type, peak intensity (PI), time to peak (TP), and area under the curve (AUC). The PI, TP, and AUC of the nodules are reported as indices by the ratio of the region of interest in the nodules to the region of interest in the thyroid parenchymal tissue.

### Histopathological Diagnosis

The histopathological results obtained after surgery were used as the only reference standard for the final diagnoses of the thyroid nodules. Patients were staged according to the eighth edition of the American Joint Committee on Cancer (AJCC)/Tumor Lymph Node Metastasis (TNM) staging system (11–13).

## Results

In this study, a total of 18 patients with 18 nodules with petal-like calcifications were summarized for their clinical, ultrasonographic and pathologic characteristics. The clinical characteristics of the 18 patients with petal-like calcifications were summarized in Table 1. After surgery, all the patients with petal-like calcifications were histopathologically confirmed as the histological classic variant PTC. A total of 18 PTC patients (four men and 14 women, age mean: 30.17 ± 7.29 y, range: 18–40 y) with petal-like calcifications were included in the analysis. Of the 18 patients, nine (50.0%) had Hashimoto thyroiditis, six (33.3%) had multiple nodules, six (33.3%) had A-TPO increased, and five (27.8%) had A-TG increased.

**Table 1:** Clinical characteristics.

**Characteristics**	***n***	**%**
**SEX**		
Male	4	22.2
Female	14	77.8
Age (years)	30.17 ± 7.29 (18–40)	
≤ 55 y	18	100
> 55 y	0	0
**MULTIFOCALITY**		
Yes	6	33.3
No	12	66.7
**HASHIMOTO THYROIDITIS**		
Yes	9	50.0
No	9	50.0
**TSH**		
Normal	18	100.0
Abnormal	0	0
**FREE THYROXINE**		
Normal	18	100.0
Abnormal	0	0
**FREE TRIIODOTHYRONINE**		
Normal	18	100.0
Abnormal	0	0
**A-TPO**		
Increased	6	33.3
Normal	12	66.7
**A-TG**		
Increased	5	27.8
Normal	13	72.2

*TSH, thyroid-stimulating hormone; A-TPO, thyroid peroxidases antibody; A-TG, thyroglobulin antibody*.

**Figure 2:**
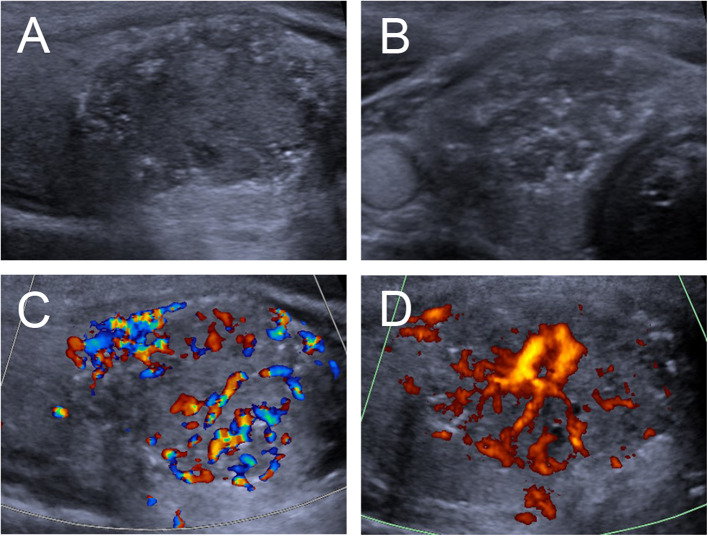
Ultrasound images of an 18-y-old female PTC patient with petal-like calcification of the right thyroid lobe. **(A)** Longitudinal and **(B)** Horizontal gray-scale sonograms; the thyroid nodules had a solid component, hypoechoic echogenicity, and ill-defined margin. **(C)** Color Doppler and **(D)** Energy Dopper sonograms showed abundant internal and peripheral vascularies.

**Figure 3:**
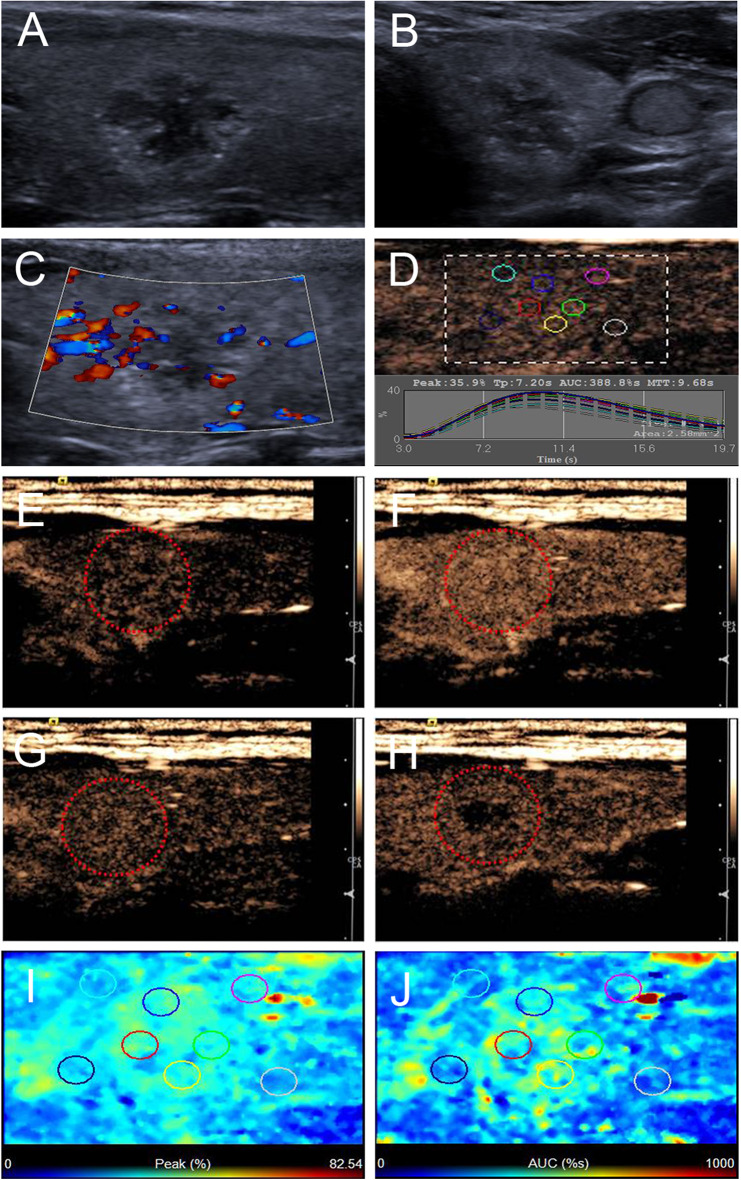
Ultrasound images of a 26-y-old female PTC patient with petal-like calcification of the left thyroid lobe. **(A)** Longitudinal and **(B)** horizontal gray-scale sonograms; the thyroid nodule had a solid component, hypoechoic echogenicity and ill-defined margin. **(C)** Color Doppler sonogram showed moderate peripheral vasculary. **(D)** TICs of the thyroid nodule and peripheral thyroid parenchyma with different regions of interest (different color circles). **(E–H)** CEUS sonograms of the thyroid nodule **(E)** at 6 s (wash-in), **(F)** 9 s (time to peak), **(G)** 15 s (wash-out), and **(H)** 33 s (wash-out), revealing diffuse and homogeneous enhancement (circle) across the whole lesion, and the center of the lesion washed out clearly at 33 s. **(I)** Parametric color map showing that peak intensity values for the nodule were partially green, and the adjacent thyroid parenchyma was blue, indicating that the peak intensity of the nodule was higher than that of the peripheral thyroid parenchyma. **(J)** Parametric color map showing that AUC values for the nodule were mixed with green and yellow, and adjacent thyroid parenchyma was blue, indicating that the AUC of the nodule was higher than that of the peripheral thyroid parenchyma.

**Figure 4:**
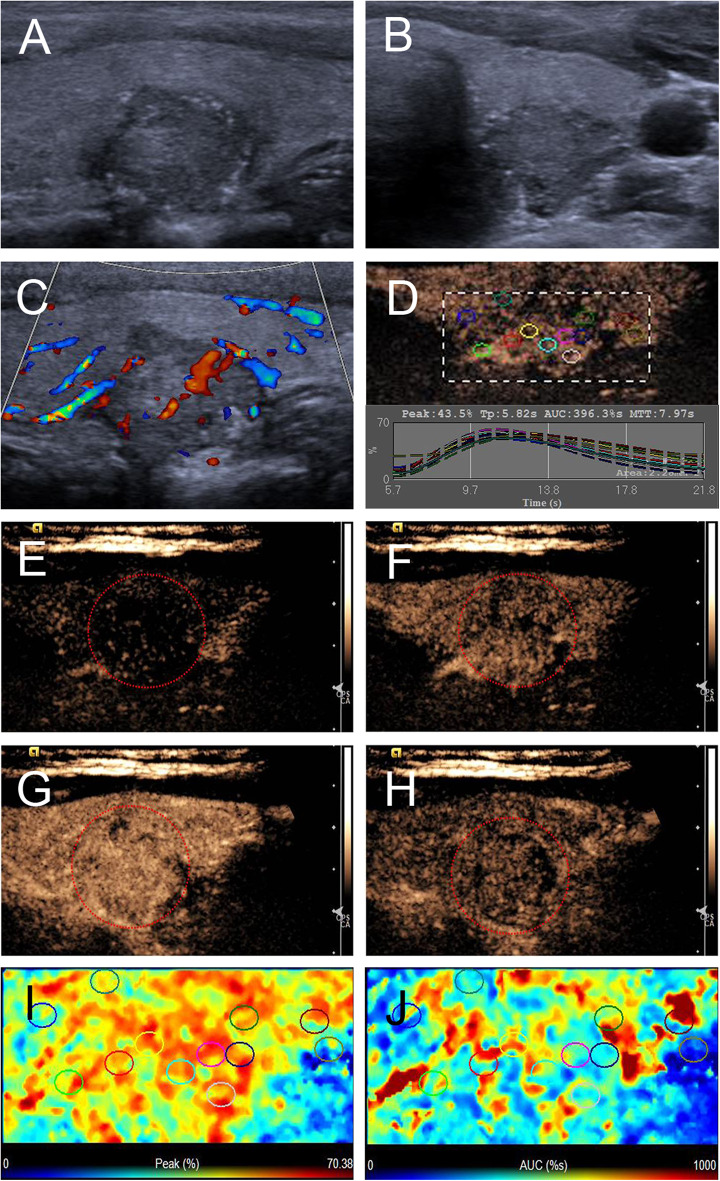
Ultrasound images of a 26-y-old female PTC patient with petal-like calcification of the left thyroid lobe. **(A)** Longitudinal and **(B)** horizontal gray-scale sonograms; the thyroid nodule had a solid component, hypoechoic echogenicity, ill-defined margin, and capsule contact with protrusion. **(C)** Color Doppler sonogram showed moderate internal and peripheral vascularies. **(D)** TICs of the thyroid nodule and peripheral thyroid parenchyma with different regions of interest (different color circles). **(E–H)** CEUS sonograms of the thyroid nodule **(E)** at 6 s (wash-in), **(F)** 9 s (wash-in), **(G)** 11 s (time to peak), and **(H)** 15 s (wash-out), revealing diffuse, and heterogeneous enhancement (circle) across the whole lesion. **(I)** Parametric color map showing that peak intensity values for the nodule were mixed with red and yellow, and adjacent thyroid parenchyma were blue and yellow, indicating that the peak intensity of the nodule was higher than that of the peripheral thyroid parenchyma. **(J)** Parametric color map showing that AUC values for the nodule were mixed with blue, yellow and red, and adjacent thyroid parenchyma were almost the same, indicating that the AUC of the nodule was equal to that of the peripheral thyroid parenchyma.

The ultrasonographic characteristics of the thyroid nodules with petal-like calcifications are outlined in Table 2. Among the 18 thyroid nodules in the 18 PTC patients, all nodules were solid in composition; only one (5.6%) had taller than wider shapes, 11 (61.1%) had ill-defined margins (Figures 2–4), 17 (94.4%) had marked hypoechoic or hypoechoic echogenicity (Figures 2–4), seven (38.9%) had acoustic posterior reinforcement (Figures 2, 3), 12 (66.7%) had capsule contact with protrusion (Figure 4), and six (33.3%) had internal vascularity (Figures 2, 4). From CEUS examination, 11 (61.1%) nodules had a hyper- or isoenhancement type (Figures 3, 4), which meant the majority of the nodules underwent a higher or equal enhancement compared with those of parenchymal tissue. Fifteen (83.3%) had a centripetal perfusion pattern (Figures 3, 4), representing most of the nodules received the perfusion of microbubbles from the periphery to the center. Twelve (66.7%) had a PI index ≥1 (Figures 3, 4), indicating that 66.7% of nodules had a higher PI than those of parenchymal tissue. Eight (44.4 %) had a TP index ≥1 (Figure 4), implying that 44.4% of nodules had a longer or equal time to peak as the parenchymal tissue. And 10 (55.6%) had an AUC index ≥1 (Figures 3, 4), showing that 55.6% of nodules had a higher AUC than those of parenchymal tissue. According to the Kwak TI-RADS classification, 18 nodules (100%) were classified as category 4c (three or four suspicious US features; high possibility of malignancy).

**Table 2:** Ultrasonographic characteristics.

**Characteristics**	**n**	**%**
**Conventional US parameters**		
**TALLER THAN WIDE SHAPE**		
Yes	1	5.6
No	17	94.4
**ILL-DEFINED MARGIN**		
Yes	11	61.1
No	7	38.9
**HYPOECHOIC ECHOGENICITY**		
Yes	17	94.4
No	1	5.6
**POSTERIOR TRANSLUCENCY**		
Yes	7	38.9
No	11	61.1
**CAPSULE CONTACT WITH PROTRUSION**		
Yes	12	66.7
No	6	33.3
**INTERNAL VASCULARITY**		
Yes	6	33.3
No	12	66.7
**CEUS parameters**		
**HYPER-OR ISO ENHANCEMENT TYPE**		
Yes	11	61.1
No	7	38.9
**CENTRIPETAL PERFUSION PATTERN**		
Yes	15	83.3
No	3	16.7
**PI INDEX** **≥1**		
Yes	12	66.7
No	6	33.3
**TP INDEX** **≥1**		
Yes	8	44.4
No	10	55.6
**AUC Index** **≥1**		
Yes	10	55.6
No	8	44.4

*US, ultrasound; CEUS, contrast-enhanced ultrasound; PI, peak intensity; TP, time to peak; AUC, area under the curve*.

The pathologic characteristics of all the patients are summarized in Table 3. Of the 18 patients, five underwent cervical central and lateral lymph node dissection, and 13 underwent central lymph node dissection. According to the eighth edition of the AJCC/TNM staging system, all patients were in TNM stage I and had no obvious distant metastases (M0 classification). The mean diameter of PTCs with petal-like calcifications was 16.72 ± 9.01 mm (range: 6–34 mm), and 13 (72.2%) patients had a tumor size > 10 mm (T1 classification). After histopathological diagnosis, five (27.8%) patients were shown to have both cervical central and lateral lymph node metastasis (N1b classification), eight (44.4%) were shown to have cervical central lymph node metastasis (N1a classification), and the last five (27.8%) had no cervical lymph node metastasis (N0 classification).

**Table 3:** Pathological characteristics according to eighth edition of AJCC/TNM classification system.

**Characteristics**	***n***	**%**
Tumor size (mm)	16.72 ± 9.01 (6–34)	
≤ 10 mm	5	27.8
> 10 mm	13	72.2
**T CLASSIFICATION**		
T1	13	72.2
T2	5	27.8
**N CLASSIFICATION**		
N0	5	27.8
N1a	8	44.4
N1b	5	27.8
**M CLASSIFICATION**		
M0	18	100
TNM stage		
I	18	100

## Discussion

High-resolution US is recommended for preoperative screening of malignant thyroid nodules from benign nodules and evaluating cervical lymph node metastasis (14–16). Previous studies have reported that PTC with cervical lymph node metastasis exhibits aggressive behavior and is associated with a poor prognosis (13, 14). As one of the more suspicious thyroid sonographic features, microcalcification has been proven to be highly predictive of central compartment lymph node metastases (17, 18). However, to our knowledge, it is rare for ultrasound to detect thyroid nodule petal-like calcifications, and their relationship with thyroid carcinoma has never been reported before.

In the present study, we collected 18 thyroid nodules with petal-like calcifications, which appeared as many scattered hyperechogenic spots around solid thyroid nodules with the appearance of flower petals, with a cystic-like dark area ahead of each hyperechogenic spot. The histopathologic result from all the patients was PTC, and 13 (72.2%) patients had cervical lymph node metastasis; however, previous studies have indicated that only 60–75% of thyroid nodules with microcalcification were PTC (4, 5), which indicates that petal-like calcifications in thyroid nodules are a fairly special sonographic characteristic of PTC and are highly associated with cervical lymph node metastasis. Fortunately, owing to the young mean age of our sample population, all patients in this study were in TNM stage I according to the eighth edition of the AJCC/TNM staging system.

According to the Kwak TI-RADS classification, five US suspicious features (solid composition, marked hypo-echogenicity or hyper-echogenicity, irregular or microlobulated margins, taller-than-wide shape, and presence of microcalcifications) were used to categorize the thyroid nodules, with TI-RADS scores of 3 (no suspicious US features), 4a (one suspicious US feature), 4b (two suspicious US features), 4c (three or four suspicious US features), and 5 (five suspicious US features) (10). In the current study, all thyroid nodules with petal-like calcifications presented with a solid composition and microcalcifications, two suspicious US features according to the Kwak TI-RADS classification guidelines from 2011 (10). In addition, the majority of these nodules had ill-defined margins and marked hypoechoic or hypoechoic echogenicity. The nodules consequently had a high TI-RADS classification due to the presence of three or four suspicious US features. Furthermore, CEUS characteristics showed that the majority of nodules had a hyper- or isoenhancement type, a centripetal perfusion pattern, a PI index ≥1 and an AUC index ≥1. Huang et al. reported that hyper- or iso-enhancement was one of the most useful US features for predicting the presence of cervical central lymph node metastasis (18), which is consistent with our results, demonstrating that the plentiful blood supply of the thyroid tumor may be associated with the aggressive biological behavior of PTC.

This study had many limitations. Firstly, all of the nodules with petal-like calcifications identified in our study ended up being PTC. So, data on whether benign thyroid nodules display such petal-like calcifications is lacking in our study. The sample size of our study population was small. Second, an unavoidable selection bias existed due to only patients who underwent surgery included in this study. A large-scale study is needed in the future to evaluate the association of petal-like calcifications with their corresponding pathogenetic characteristic.

## Conclusions

In conclusion, we first reported a special pattern of calcification in thyroid nodules, petal-like calcifications, which only occurred in malignant nodules with a high incidence of lymph node metastasis. This kind of calcification appears as numerous scattered hyperechogenic spots around solid thyroid nodules with the appearance of flower petals, with a cystic-like dark area ahead of each hyperechogenic spot in ultrasonographic images, which is called black-and-white foci. In addition, the majority of these nodules had ill-defined margins, marked hypoechoic or hypoechoic echogenicity, capsule contact with protrusion, a hyper- or isoenhancement type, a centripetal perfusion pattern, a PI index ≥1 and an AUC index ≥1. Therefore, petal-like calcifications in thyroid nodules may be a specific ultrasonographic feature associated with the aggressive biological behavior of PTC.

## Data Availability Statement

The raw data supporting the conclusions of this article will be made available by the authors, without undue reservation, to any qualified researcher.

## Ethics Statement

The studies involving human participants were reviewed and approved by the ethics committee of the Second Xiangya Hospital of Central South University. The patients/participants provided their written informed consent to participate in this study. Written informed consent was obtained from the individual(s) for the publication of any potentially identifiable images or data included in this article.

## Author Contributions

CN contributed to the conception and design of the work. QP and CN participated to data analysis and manuscript writing. QZ and SC participated to data collection and patient follow-up.

## Conflict of Interest

The authors declare that the research was conducted in the absence of any commercial or financial relationships that could be construed as a potential conflict of interest.
